# Integrating electronic health records with other data sources for postmarket drug safety signal identification: a review

**DOI:** 10.3389/fdsfr.2024.1428831

**Published:** 2024-11-13

**Authors:** Julie V. Kim, Sharon E. Davis, Michael E. Matheny, Joshua C. Smith

**Affiliations:** ^1^ Department of Biomedical Informatics, Vanderbilt University Medical Center, Nashville, TN, United States; ^2^ Geriatric Research, Education, and Clinical Center (GRECC), VA Tenneesee Valley Healthcare System, Nashville, TN, United States

**Keywords:** pharmacovigilance, safety signal identification, electronic health records, spontaneous reporting systems, adverse drug events

## Abstract

Electronic health records (EHRs) have emerged as resources for both the identification of adverse drug events (ADEs) and general population health surveillance, however questions remain around how best to utilize EHR data for drug safety signal identification. While the majority of signal identification research has utilized spontaneous reports and health insurance claims, these data also have limitations. Pharmacovigilance approaches combining EHR data with other data sources have the potential to address many of the shortcomings of individual sources. This mini-review seeks to provide an overview of some of the recent research leveraging EHR data in combination with spontaneous reports, claims data, and other pharmacovigilance data sources for drug safety signal identification. Studies have shown that combining EHR data with these and other sources is often beneficial compared to the use of a single source alone, however the synergism or friction introduced is insufficiently explored in current literature. Our review explores how EHR data benefits signal identification when used in combination with other sources, what methods have been applied, and what considerations have previously been noted. Finally, we identify gaps in current research and highlight important considerations for future work using multiple real world data sources for drug safety surveillance.

## 1 Introduction

Electronic health records (EHRs) have emerged as resources for both the identification of adverse drug events (ADEs) and general population health surveillance (Desai et al., 2021). However, as we have shown in previous work, questions remain around how best to utilize EHR data for drug safety signal identification, particularly with regards to 1) challenges in leveraging multiple data domains in the EHR in concert, 2) challenges in appropriately merging EHR and non-EHR data sources together, and 3) methods for effectively analyzing data under these different data collection strategies (Davis et al., 2023).

Signal identification studies using EHR data alone have largely focused on a limited set of data domains, such as structured diagnosis codes, laboratory results, or free-text clinical notes. Most studies have not combined these EHR domains together, or explored other components within EHRs (Davis et al., 2023). And despite these rich data, the extent to which studies adjust for confounding variables has been limited and inconsistent. Consequently, discovered ADE signals may fluctuate in strength of evidence and validity.

While the use of EHR data for safety signal identification is promising, the majority of signal identification research has utilized spontaneous reporting systems (SRS) and health insurance claims data (Lucas et al., 2022). Spontaneous reports have long served as the primary vehicle for post-market drug safety signal identification. Available in SRS such as the FDA Adverse Event Reporting System (FAERS) and the European Medicines Agency’s EudraVigilance, spontaneous reports detail suspected adverse events related to drugs, biologics, and other medicinal products (Postigo et al., 2018; Center for Drug Evaluation and Research, 2019). SRS are particularly useful for detecting unexpected and unknown adverse events where the occurrence would be expected to be zero or near zero (Pal et al., 2013).

Over the last decade, health insurance claims data, such as that available through the FDA Sentinel System, have served as another primary data source for safety surveillance (Huang et al., 2014). The use of claims data addresses many of the shortcomings of SRS, by allowing ascertainment of cohorts of patients, longitudinal patient conditions, diagnostics, and treatments over time. These distinctly different data sources in turn support a different family of methods to adjust for patient clinical states.

There are also emerging novel data sources that are being explored for their relevance in drug safety surveillance, such as social media databases (Lee et al., 2021), internet search trends (White et al., 2014), and patient reported outcomes (Habib et al., 2021). These data sources represent distinctly different adverse effect information and may provide useful data such as the subjective experience of patients in their own words. Studies using these unconventional data sources have been able to duplicate known signals, however relatively few studies exist compared to those utilizing traditional data.

While each of these data sources have advantages, they also each have significant challenges. For example, SRS suffer from a number of well-known limitations, such as the passive nature of their surveillance (reporters must suspect an association between a medication and outcome and then make the effort to report it), chronic under-reporting, and a lack of linked longitudinal patient information which limits the ability to control for confounding effects (Hazell and Shakir, 2006; Palleria et al., 2013). Claims data are limited by a narrow range of data domains, lacking laboratory test data, radiology data, and clinical text. Social media are limited by challenges in parsing unstructured and nonmedical reporting of symptoms and events into data representations amenable to surveillance (Rees et al., 2018). In addition, linking social media data to more traditional medical data sources is particularly difficult.

Lastly, EHR data also have limitations, being noisier and more variable in data collection practices. Compared to SRS, however, the EHR adds robust, longitudinal data with the potential to better control for confounders, as well as a better representation of prevalence in a population. Compared to claims data, the EHR adds more detailed and nuanced patient information in the form of vital signs, laboratory results, and unstructured clinical text, describing details that may not be captured in structured codes.

Pharmacovigilance approaches combining these other data sources with EHR data have the potential to address the shortcomings of individual sources. This review seeks to provide an overview of some of the recent research leveraging EHR data in combination with other data sources for drug safety signal identification. As there are limited examples of such research, we also highlight research gaps and potential future directions in this field. Our overall objective is to discuss how these data sources have been used together in the past to further elucidate how they can best complement one another to improve drug safety surveillance in the future.

## 2 Usage of EHRs with disparate data sources for signal identification

**FIGURE 1:**
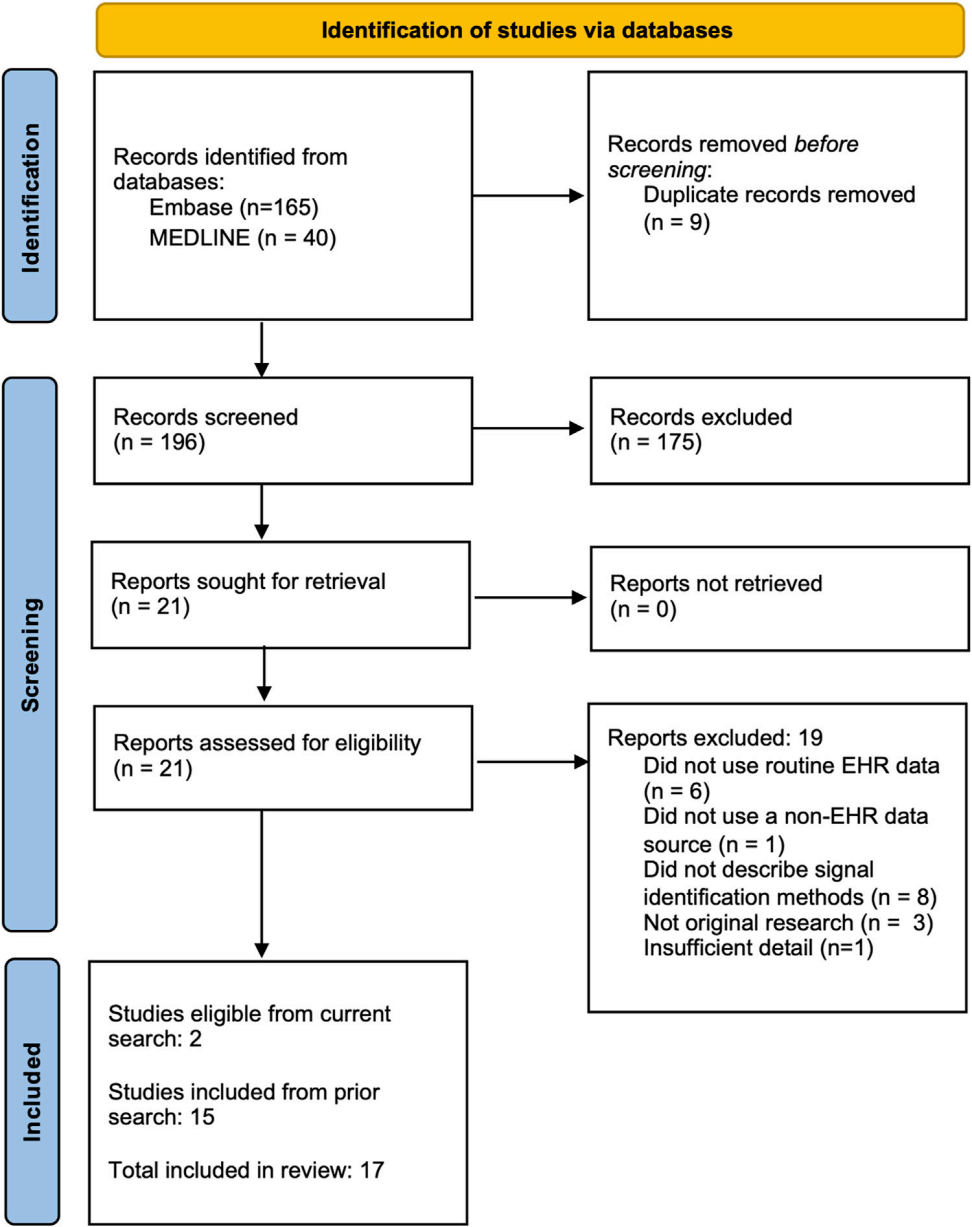
PRISMA Flow Diagram of search results.

We conducted a scoping review to understand the current state of postmarketing medication surveillance efforts using EHR data in combination with disparate data sources. Using a search methodology previously developed to identify studies using EHR for signal identification (Davis et al., 2023), we searched for citations encompassing each of three relevant domains -- pharmacovigilance biomedical research, analytic methods used for signal identification, and EHR-based data resources (search queries in Supplemental Materials). We revised this search methodology with additional eligibility criteria to focus on the use of EHR alongside non-EHR data and updated the search results with relevant publications in MEDLINE and EmBase indexed through 20 March 2024. Studies were eligible for inclusion in this review if they 1) reported original research; 2) conducted analyses to identify excess adverse outcomes associated with a medication regardless of whether a link between the medication and outcome were noted at the time of the event; and 3) analyzed both patient-level EHR data and data from at least one other source. From our prior review, 15 studies were eligible; our updated search found 196 additional studies, 21 of which were retained after initial abstract screening, and 2 of which passed full text review. Combing the original and updated search, data was extracted from a total of 17 studies (see Figure 1).

We note that a key limitation of our search strategy was that it required mentions of EHR data specifically, as well as another separate source. Results therefore did not necessarily include studies in which data were previously integrated (i.e., linked on the patient or visit level). For example, integrated care organizations, such as Kaiser Permanente in the United States or public health services in many countries, integrate health insurance with medical services. Similarly, commercial resources, such as those provided by TriNetX, combine data sourced from EHRs and insurance claims into a single database (TriNetX, 2024). Despite data from these institutions including longitudinal claims and detailed EHR data, few of these studies classified themselves as clearly using EHR data in either titles or abstracts. For this reason, few studies utilizing claims data linked to EHR data were identified here.

**FIGURE 2:**
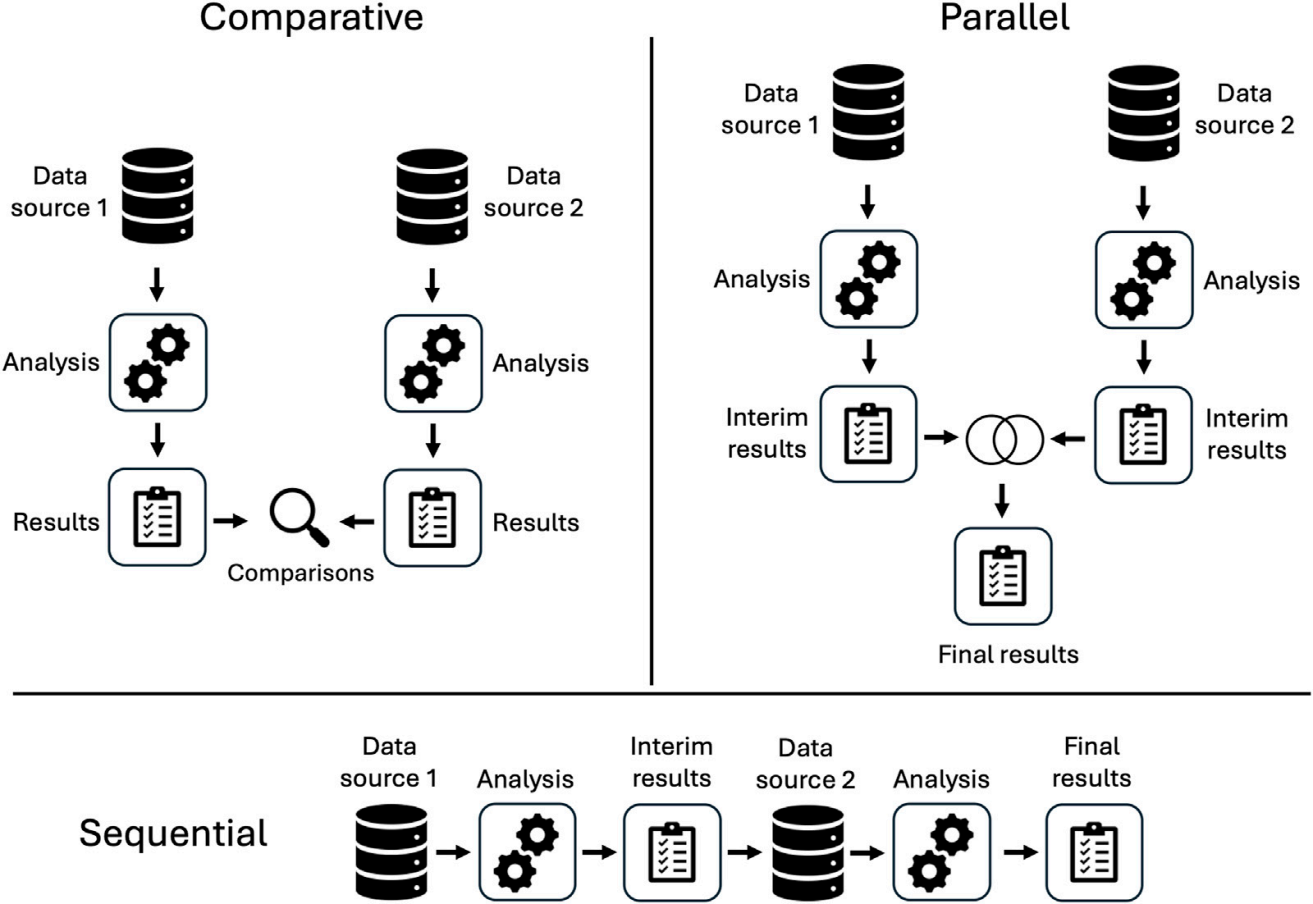
Analytic pipelines for signal detection in disparate data sources.

An overview of included studies is presented in Table 1. Spontaneous reporting system data was the non-EHR data source in 16 of the 17 studies. Two studies considered claims data. Most studies included minimal or no control for confounding and applied variations of disproportionality analysis (DPA). We classified the analytic pipelines used to integrate information across data sources into three categories: comparative, parallel, or sequential (see Figure 2). In seven studies, comparative analyses of EHR data and the alternative data source were used for evaluation purposes only rather than to enhance information and insight across data sources. Four studies conducted parallel analyses in the EHR and alternative data source, combining signals identified in each or conducting meta-analyses to integrate separate findings. Six studies developed a sequential pipeline in which the results of signal detection in one data source informed the ADEs assessed in the other. With one exception (Shin et al., 2021), these studies first evaluated spontaneous reports and used EHR data to confirm the ADE signals. Overall, studies found no single source consistently provided the best evidence for ADE signal identification, highlighting the potential utility of considering multiple sources of information.

**TABLE 1:** Details of included studies.

Study	EHR data	Non-EHR data	Data Types	Data Source Integration	Control for confounding	Analytic approach	Key findings
Trifirò et al. (2011)	1996–2010, European multiple national datasets (EU-ADR)	1968+, SRS (FAERS and VigiBase)	EHR data: structured data on medications and diagnoses Non-EHR data: structured data on medications and diagnoses	Separate analyses for comparison	None	EHR: DPA with LGPS; SRS: (EBGM)	More drug-event pairs could be explored in SRS for those events suspected to be ADEs, but EHRs revealed more potential signals and may reveal ADEs not commonly reported for adverse events that are perceived as common in general populations
Harpaz et al. (2013)	2004–2010, US academic medical center	1968–2010, SRS (FAERS)	EHR data: structured laboratory data and unstructured data from discharge summaries, admission notes, and outpatient office visit notes Non-EHR data: structured data on medications and diagnoses	Parallel analysis (intersection of results)	None	DPA (MGPS for SRS and OR for EHR)	Combined system best for finding emerging ADE signals where there may be less consistent reporting
Iyer et al. (2014)	1994–2012, US academic medical center	1997–2012, SRS (FAERS)	EHR data: unstructured data from both inpatient and outpatient notes, including radiology, pathology, and transcription reports Non-EHR data: structured data on medications and diagnoses	Separate analyses for comparison	EHR: PS matching age, sex, race, note count, diagnoses count, medication count	DPA for ORs (EHR) and DPA with MGPS (SRS)	Comparable performance from EHR and SRS, not used together, only for comparison. Could find some in EHR before first report to FAERS
Patadia et al. (2015)	1995–2010, European multiple national datasets (EU-ADR)	1969+, SRS (FAERS)	EHR data: structured data on medications and diagnoses Non-EHR data: structured data on medications and diagnoses	Separate analyses for comparison	None	EHR: DPA with LGPS; SRS: (GPS)	Both had high specificity. EHRs were better at detecting signals when only data prior to regulatory action was used; in SRS signals were not as strong before regulatory action -- suggesting reporting patterns and use patterns in the two datasets matter
Li et al. (2015)	2004–2010, US academic medical center and large data consortium	2004–2010, SRS (FAERS); Claims (MarketScan)	EHR data: structured data on diagnoses and medications, as well as unstructured data from admission notes and discharge summaries Non-EHR data (SRS): medication data from unstructured case reports and structured data on diagnoses Non-EHR data (claims): structured data on medications and diagnoses	Parallel analysis (combined through meta-analysis)	Smaller EHR and FAERS adjusted for other medication use; Larger EHR and claims used self-controlled case series	Smaller EHR and FAERS used regression for ORs, Claims and larger EHR preprocessed with various DPA methods	Large scale EHR or claims data in combination with SRS improved detection of ADE, but small scale EHR did not
Pacurariu et al. (2015)	2000–2010, European multiple national datasets (EU-ADR)	2000–2010, SRS (EudraVigilance)	EHR data: structured data on medications and diagnoses Non-EHR data: structured data on medications and diagnoses	Separate analyses for comparison	None	EHR: DPA with LGPS; SRS: DPA with PRR	EHR may be particularly useful for more common events, but SRS may generally be more cost effective in terms of number needed to detect
Star et al. (2015)	Through 2011, European national dataset	2011, SRS (VigiBase)	EHR data: structured data on medications and diagnoses Non-EHR data: structured data on medications and diagnoses	Sequential analyses (SRS results informed EHR analyses)	None	SRS: DPA and manual review; EHR: temporal pattern analysis with chronographs	Lack of power for analyses EHR for several ADEs highlighted in SRS due to THIN being outpatient data (some meds only inpatient) and some meds not being widely used in United Kingdom
Lorberbaum et al. (2016a)	1996–2014, US academic medical center	*** SRS (TWOSIDES)	EHR data: structured data on demographics and medications, ECG report elements Non-EHR data: structured coded data on medications and diagnoses	Sequential analyses (SRS results informed EHR analyses)	EHR: stratification by sex and race	SRS: DPA with binary outcome; EHR: Mann-Whitney Y test for QTc interval; Signal corroborated in the EHR were further evaluated in lab studies	Identified potential DDI and documented evidence in lab that may explain mechanism
Lorberbaum et al. (2016b)	US academic medical center	2004–2009, SRS (FAERS)	EHR data: structured data on demographics, diagnoses and medications, ECG reports Non-EHR data: structured coded data on medications and diagnoses	Sequential analyses (SRS results informed EHR analyses)	EHR: stratification by sex and adjustment for co-medications	SRS: logistic regression; EHR: Mann-Whitney Y test for QTc interval	Used indirect outcome fingerprints from FAERS and confirmed with more accurate outcome ascertainment in EHR
Wang et al. (2017)	1998–2013, US academic medical center	2004–2011, SRS (FAERS)	EHR data: medication and diagnoses data extracted from clinical unstructured narratives (note types not specified) Non-EHR data: Structured diagnoses data and medication data extracted from unstructured reports	Sequential analyses (SRS results informed EHR analyses)	None	DPA	Demonstrated framework for signal discovery in SRS and subsequent signal validation in EHR
Patadia et al. (2018)	1995–2010, European multiple national datasets (EU-ADR)	1968–2010, SRS (VigiBase)	EHR data: structured data on medications and diagnoses Non-EHR data: structured data on medications and diagnoses	Separate analyses for comparison	None	EHR: DPA with LGPS; SRS: (GPS)	Earlier detection in EHR data than SRS. Some reporting to SRS of events prior to warnings but reported after
Wang et al. (2018)	1995–2017, US academic medical center	SRS (FAERS)	EHR data: medication and diagnoses data extracted from clinical unstructured narratives (note types not specified) Non-EHR data: Structured diagnoses data and medication data extracted from unstructured reports	Separate analyses for comparison and parallel analysis (combination)	EHR: self-controlled series	DPA with ORs	Significant overlap of findings from both sources, combining improved recall
Yu et al. (2020)	2004–2018, US academic medical center	2004–2018, SRS (FAERS)	EHR data: structured data on medications and diagnoses Non-EHR data: structured data on medications and diagnoses	Separate analyses for comparison	None	DPA	In monthly repeated analyses, ADEs may be detectable earlier in EHR data than in SRS
Akimoto et al. (2021)	2004–2020, Japanese academic medical center	2004–2020, SRS (JADER)	EHR data: structured data on demographics, labs, medications and diagnoses Non-EHR data: structured data on demographics, medications and diagnoses	Separate analyses for comparison	Both: adjustment for age, sex, medications, diagnoses	Logistic regression	Higher OR estimates from SRS compared to EHR (“may be overestimated due to the existence of reporting bias.”
Shin and Lee (2021)	2014–2018, South Korean academic medical center	2012–2018, SRS (FAERS)	EHR data: structured data on demographics, medications and diagnoses, as well as pure tone audiometry reports Non-EHR data: structured data on demographics, medications and diagnoses	Parallel analysis (combined through meta-analysis)	EHR: PS matching based on age, sex, utilization	EHR: Cox regression, SRS: DPA with OR	Identified novel potential ADE for further evaluation
Shin et al. (2021)	2005–2011, South Korean academic medical center	2013–2017, SRS (KAERS)	EHR data: structured data on demographics, medications and diagnoses Non-EHR data: structured data on demographics, medications and diagnoses	Sequential analyses (EHR results informed SRS analyses)	None	EHR: Cox regression; SRS DPA with ROR	Note: KAERS not all SRS; Many more potential ADE identified in EHR than in SRS, particularly due to small reporting numbers for some drugs
Kim et al. (2024)	2012–2019, South Korean academic medical center	2017–2019, Claims (HIRA)	EHR data: structured data on demographics, medications and diagnoses, as well as unstructured clinical text such as nursing records and progression notes Non-EHR data: structured data on medications and diagnoses	Sequential analyses (Claims results informed EHR analyses)	EHR: Matching on age, sex, diagnosis	Cox regression and DPA	Claims analyses identified known and novel ADEs, Novel ADEs validated in EHR against diagnosis code AE and text mining AE which led to different results

Several studies noted important considerations when using both EHR and spontaneous reporting data for observational postmarketing surveillance. First, the timing of analyses and public concern over ADEs influences data collection in both EHR and spontaneous reporting data, with implications for the ability to detect signals. For example, while several studies found that ADEs could be found in EHR data earlier than in spontaneous reports, the issuing of public warnings or publications of studies reporting suspected ADEs impact the data available in each source (Iyer et al., 2014; Patadia et al., 2018; Yu et al., 2020). After awareness of a suspected ADE, more spontaneous reports may be submitted even for events occurring before the warning and the drug-event pair of interest may be less commonly observed in EHR data as fewer patients at risk may be treated with the drug (Patadia et al., 2015). As a consequence, while ADE signals may be easier to identify in EHR data prior to the warning, afterward the same ADE signals may become harder to identify in EHR data and easier to identify in spontaneous reports (Patadia et al., 2015; Patadia et al., 2018). A second consideration is the size of the observational EHR data and the population covered. Small EHRs and systems covering populations unlikely to receive a particular drug may not provide sufficient information for analysis (Li et al., 2015; Star et al., 2015). Finally, for more common adverse events, EHR data may be a particularly useful data source as healthcare providers and patients may be less likely to consider an association between a drug exposure and the event, making spontaneous reports unlikely to be filed (Trifirò et al., 2011; Pacurariu et al., 2015).

One unique advantage of EHRs over most other data sources is the availability of unstructured clinical documents. While these documents often contain a richer, detailed, and more nuanced view of the patient, their lack of structure requires the use of natural language processing (NLP) to extract useable information. Out of the 17 studies we reviewed, 6 utilized this unstructured text. This includes EHR data such as admission notes, discharge summaries, outpatient visits notes, radiology and pathology reports, nursing records, and inpatient progress notes; non-EHR data sources included free-text adverse event reports. Several studies utilized standard biomedical tools such as MedLEE (Harpaz et al., 2013) and Medtagger (Wang et al., 2017; Wang et al., 2018) for extracting outcomes from text, as well as MedEx and MedXN for medication extraction (Wang et al., 2017; Wang et al., 2018). Other studies employed custom scripts, regular expressions, and text searching for extracting relevant data (Harpaz et al., 2013; Iyer et al., 2014; Kim et al., 2024; Li et al., 2015). Nearly all these studies mapped extracted concepts to vocabularies within or subsets of the Unified Medical Language System (UMLS).

## 3 Trends and questions arising from prior research

Most studies found in our review investigated the combination of EHR data with spontaneous reports. Few identified studies combined EHR data with health insurance claims data (since those studies more often utilized integrated data sources and were not included, a limitation described above). We found no studies that combined social media data with EHR data. Combining SRS with EHR data has been shown to improve both accuracy and recall for signal identification (Harpaz et al., 2013; Wang et al., 2018). Despite this, combining two or more disparate sources is not a widespread practice and the exact benefits have not been fully explored. It is also important to emphasize that we excluded combinations of sources that did not involve the EHR, for example, spontaneous reports and claims, or spontaneous reports with social media. These areas likely also need further investigation and may benefit from independent reviews.

Different pipelines have also been explored, but not directly compared. For most studies classified as sequential (when the results from one source informed analysis of the other), the SRS was used to identify signals that were further validated in the EHR; in fewer studies, the reverse was done, wherein signals from EHR data informed analysis of spontaneous reports. Comparative studies may allow us to identify strengths and weaknesses of each approach, but it is currently unknown how different methods of directionality and/or integration of two data sources may influence the signals identified. What is known is that when integrating data sources, it is important to ensure they are comparable. For example, usage rates for a medication of interest may differ significantly between one source and another if the data is sourced from different countries or regions, or if they encompass different patient populations (Star et al., 2015).

Regarding statistical methods, most combination EHR studies used disproportionality analysis methods to identify signals. It is uncertain whether certain methods are more effective at reducing noise and identifying signals as there have been only a few studies directly comparing methods in a single data source (Park et al., 2020; Khouri et al., 2021). There remains a need for further comparison of methods to test which one may be more effective or feasible to use for signal identification in two different data sources, or when one method may be preferred over another. Importantly, there was little to no adjustment for confounding variables when combining the EHR with another data source for signal identification. The EHR can be a vast resource with significant depth and nuance, but not all EHR variables are regularly utilized. While some studies adjusted for confounding using propensity scores, age, sex, or medications, the majority described no control for confounding (see Table 1). This remains consistent with findings on the lack of confounding variables when utilizing EHR data alone (Davis et al., 2023). Selecting confounding features and appropriately adjusting for their influence may be complex and require tailored approaches (Wang et al., 2021). Further, it may be a difficult task to uniformly adjust for confounding variables across two data sources. Best practices for controlling for confounding in EHR-based signal identification warrant further research.

## 4 Future directions

As with the use of EHR data alone, many signal identification methods have been applied when using combined sources. Data from EHRs may be more useful for detection of novel ADEs, but additional evaluation of methods may identify which sources are better for particular scenarios, such as with rare or severe ADEs. Further adjustment for confounding may also reduce noise in the signals and make use of the myriad types of data within the EHR. Benchmarking studies may help expand knowledge about the use of the EHRs with other data sources. Shared reference standards with detailed definitions of exposures and outcomes would allow researchers to systematically investigate the performance of different signal identification methods with different combinations of data sources, analytic pipelines, and controls for confounding features. Research should consider factors such as the rarity of the adverse event, severity of the adverse event, and reporting patterns, among other variables. Various existing reference standards may be suitable for assessing the use of combined data sources, but further study may be necessary.

Similarly, the data sources used, and the order in which they are analyzed, can also impact which signals are identified or prioritized. As described above, the utility of pharmacovigilance data is also influenced by the publication of research or warnings surrounding an ADE. Prior to suspicion surrounding an association, one data source may be better suited for identifying safety signals; after warnings are issued, another source may be more reliable. Awareness of this influence can be used to improve signal identification using the integration of multiple sources and warrants exploration of potential adjustment or changes in analytic design before and after public reporting of adverse events.

Disproportionality analysis methods were applied in most of our reviewed studies, but statistical, data mining, and machine learning methods should also be assessed for use with multiple-source approaches. Use of unstructured text data in the reviewed studies consisted primarily of concept recognition using rule-based NLP approaches. Modern techniques such as deep learning or large language models (LLMs) are a largely unexplored space and likely to offer new capacities for signal identification. For example, LLMs could support analysis of confounding factors, improved annotation of clinical text, and better information extraction from notes, adverse event reports, case studies, and other knowledge sources (Matheny et al., 2024). However, significant effort will be required to ensure LLMs support the necessary rigor required for safety signal identification.

Finally, we found only one study combining more than two sources of data on drug exposures and outcomes (Li et al., 2015). Future work integrating spontaneous reports, claims data, EHR data, and social media might prove beneficial. Additional resources such as public health databases, patient reported outcomes, and the published literature should also be considered. However, it is also important to note that combining data sources is complex, and not a panacea for pharmacovigilance. For example, sequential and parallel studies may underperform when the data source represents highly disparate populations, and integrated studies that merge data may be dependent on direct overlap between populations. Additionally, utilizing health insurance claims improves longitudinal coverage of patients, but in countries such as the United States many patients change insurance providers and health systems frequently. Many patients may not have the same access to healthcare, and instead may turn to social media in search of answers. No single source of data is complete, but multiple sources, corroborating and complementing one another, could benefit pharmacovigilance and lead to improved safety surveillance.

## 5 Conclusion

In summary, although a wide selection of computational methods has been applied to EHR and non-EHR data, it remains unclear how these methods compare to one another and the circumstances under which one method might be preferred over another in the setting of different data source configurations. There remains a need for further research in best practices for guidance regarding selection of methods for hypothesis generation, detection, and confirmation of drug events in observational EHR data alone and in concert with other health-related data sources.

The key opportunity spaces identified in this review to pursue safety surveillance are 1) evaluation of the utility and feasibility of integrating more than two data sources together, 2) robust evaluation of the impacts on validity and variability of results from increasingly robust confounder adjustment, 3) exploring health-related and health-adjacent data sources for novel opportunities for signal identification, and 4) evaluating novel data science methods such as deep learning and large language models for both information extraction and signal identification purposes.

Capturing a patient’s journey through healthcare remains challenging even when using multiple sources, but the integration of data sources along with careful evaluation of the appropriateness, utility, and accuracy of statistical methods to conduct signal identification in these data have the potential to substantially impact utility of real-world data in drug safety signal identification efforts.

## References

[B1] Akimoto H. Nagashima T. Minagawa K. Hayakawa T. Takahashi Y. Asai S. (2021). Signal detection of potential hepatotoxic drugs: case-control study using both a spontaneous reporting system and electronic medical records. Biol. Pharm. Bull. 44, 1514–1523. 10.1248/bpb.b21-00407 34602560

[B2] Center for Drug Evaluation and Research (2019). Questions and answers on FDA’s adverse event reporting system (FAERS). Silver Spring, MD: FDA. Available at: https://www.fda.gov/drugs/surveillance/questions-and-answers-fdas-adverse-event-reporting-system-faers (Accessed May 3, 2024).

[B3] Davis S. E. Zabotka L. Desai R. J. Wang S. V. Maro J. C. Coughlin K. (2023). Use of electronic health record data for drug safety signal identification: a scoping review. Drug Saf. 46, 725–742. 10.1007/s40264-023-01325-0 37340238 PMC11635839

[B4] Desai R. J. Matheny M. E. Johnson K. Marsolo K. Curtis L. H. Nelson J. C. (2021). Broadening the reach of the FDA Sentinel system: a roadmap for integrating electronic health record data in a causal analysis framework. NPJ Digit. Med. 4, 170. 10.1038/s41746-021-00542-0 34931012 PMC8688411

[B5] Habib B. Tamblyn R. Girard N. Eguale T. Huang A. (2021). Detection of adverse drug events in e-prescribing and administrative health data: a validation study. BMC Health Serv. Res. 21, 376. 10.1186/s12913-021-06346-y 33892716 PMC8063436

[B6] Harpaz R. Vilar S. Dumouchel W. Salmasian H. Haerian K. Shah N. H. (2013). Combing signals from spontaneous reports and electronic health records for detection of adverse drug reactions. J. Am. Med. Inf. Assoc. JAMIA 20, 413–419. 10.1136/amiajnl-2012-000930 PMC3628045 23118093

[B7] Hazell L. Shakir S. A. W. (2006). Under-reporting of adverse drug reactions: a systematic review. Drug Saf. 29, 385–396. 10.2165/00002018-200629050-00003 16689555

[B8] Huang Y.-L. Moon J. Segal J. B. (2014). A comparison of active adverse event surveillance systems worldwide. Drug Saf. 37, 581–596. 10.1007/s40264-014-0194-3 25022829 PMC4134479

[B9] Iyer S. V. Harpaz R. LePendu P. Bauer-Mehren A. Shah N. H. (2014). Mining clinical text for signals of adverse drug-drug interactions. J. Am. Med. Inf. Assoc. JAMIA 21, 353–362. 10.1136/amiajnl-2013-001612 PMC3932451 24158091

[B10] Khouri C. Nguyen T. Revol B. Lepelley M. Pariente A. Roustit M. (2021). Leveraging the variability of pharmacovigilance disproportionality analyses to improve signal detection performances. Front. Pharmacol. 12, 668765. 10.3389/fphar.2021.668765 34122089 PMC8193489

[B11] Kim E. J. Kim Y.-J. Heo J. Y. Kim M. Lee S. Seo S. (2024). Detection and evaluation of signals for immune-related adverse events: a nationwide, population-based study. Front. Oncol. 13, 1295923. 10.3389/fonc.2023.1295923 38344142 PMC10854742

[B12] Lee J.-Y. Lee Y.-S. Kim D. H. Lee H. S. Yang B. R. Kim M. G. (2021). The use of social media in detecting drug safety-related new black box warnings, labeling changes, or withdrawals: scoping review. JMIR Public Health Surveill. 7, e30137. 10.2196/30137 34185021 PMC8277336

[B13] Li Y. Ryan P. B. Wei Y. Friedman C. (2015). A method to combine signals from spontaneous reporting systems and observational healthcare data to detect adverse drug reactions. Drug Saf. 38, 895–908. 10.1007/s40264-015-0314-8 26153397 PMC4579260

[B14] Lorberbaum T. Sampson K. J. Chang J. B. Iyer V. Woosley R. L. Kass R. S. (2016a). Coupling data mining and laboratory experiments to discover drug interactions causing QT prolongation. J. Am. Coll. Cardiol. 68, 1756–1764. 10.1016/j.jacc.2016.07.761 27737742 PMC5082283

[B15] Lorberbaum T. Sampson K. J. Woosley R. L. Kass R. S. Tatonetti N. P. (2016b). An integrative data science pipeline to identify novel drug interactions that prolong the QT interval. Drug Saf. 39, 433–441. 10.1007/s40264-016-0393-1 26860921 PMC4835515

[B16] Lucas S. Ailani J. Smith T. R. Abdrabboh A. Xue F. Navetta M. S. (2022). Pharmacovigilance: reporting requirements throughout a product’s lifecycle. Ther. Adv. Drug Saf. 13, 20420986221125006. 10.1177/20420986221125006 36187302 PMC9520146

[B17] Matheny M. E. Yang J. Smith J. C. Walsh C. G. Al-Garadi M. A. Davis S. E. (2024). Enhancing postmarketing surveillance of medical products with large language models. JAMA Netw. Open 7, e2428276. 10.1001/jamanetworkopen.2024.28276 39150707

[B18] Pacurariu A. C. Straus S. M. Trifirò G. Schuemie M. J. Gini R. Herings R. (2015). Useful interplay between spontaneous ADR reports and electronic healthcare records in signal detection. Drug Saf. 38, 1201–1210. 10.1007/s40264-015-0341-5 26370104 PMC4659852

[B19] Pal S. N. Duncombe C. Falzon D. Olsson S. (2013). WHO strategy for collecting safety data in public health programmes: complementing spontaneous reporting systems. Drug Saf. 36, 75–81. 10.1007/s40264-012-0014-6 23329541 PMC3568200

[B20] Palleria C. Leporini C. Chimirri S. Marrazzo G. Sacchetta S. Bruno L. (2013). Limitations and obstacles of the spontaneous adverse drugs reactions reporting: two “challenging” case reports. J. Pharmacol. Pharmacother. 4, S66–S72. 10.4103/0976-500X.120955 24347986 PMC3853673

[B21] Park G. Jung H. Heo S.-J. Jung I. (2020). Comparison of data mining methods for the signal detection of adverse drug events with a hierarchical structure in postmarketing surveillance. Life Basel Switz. 10, 138. 10.3390/life10080138 PMC7460123 32764444

[B22] Patadia V. K. Schuemie M. J. Coloma P. Herings R. van der Lei J. Straus S. (2015). Evaluating performance of electronic healthcare records and spontaneous reporting data in drug safety signal detection. Int. J. Clin. Pharm. 37, 94–104. 10.1007/s11096-014-0044-5 25488315

[B23] Patadia V. K. Schuemie M. J. Coloma P. M. Herings R. van der Lei J. Sturkenboom M. (2018). Can electronic health records databases complement spontaneous reporting system databases? A historical-reconstruction of the association of rofecoxib and acute myocardial infarction. Front. Pharmacol. 9, 594. 10.3389/fphar.2018.00594 29928230 PMC5997784

[B24] Postigo R. Brosch S. Slattery J. van Haren A. Dogné J.-M. Kurz X. (2018). EudraVigilance Medicines safety database: publicly accessible data for research and public health protection. Drug Saf. 41, 665–675. 10.1007/s40264-018-0647-1 29520645 PMC5990579

[B25] Rees S. Mian S. Grabowski N. (2018). Using social media in safety signal management: is it reliable? Ther. Adv. Drug Saf. 9, 591–599. 10.1177/2042098618789596 30283627 PMC6166319

[B26] Shin H. Cha J. Lee Y. Kim J.-Y. Lee S. (2021). Real-world data-based adverse drug reactions detection from the Korea Adverse Event Reporting System databases with electronic health records-based detection algorithm. Health Inf. J. 27, 14604582211033014. 10.1177/14604582211033014 34289723

[B27] Shin H. Lee S. (2021). An OMOP-CDM based pharmacovigilance data-processing pipeline (PDP) providing active surveillance for ADR signal detection from real-world data sources. BMC Med. Inf. Decis. Mak. 21, 159. 10.1186/s12911-021-01520-y PMC8130307 34001114

[B28] Star K. Watson S. Sandberg L. Johansson J. Edwards I. R. (2015). Longitudinal medical records as a complement to routine drug safety signal analysis. Pharmacoepidemiol. Drug Saf. 24, 486–494. 10.1002/pds.3739 25623045 PMC5024044

[B29] Trifirò G. Patadia V. Schuemie M. J. Coloma P. M. Gini R. Herings R. (2011). EU-ADR healthcare database network vs. spontaneous reporting system database: preliminary comparison of signal detection. Stud. Health Technol. Inf. 166, 25–30. 10.3233/978-1-60750-740-6-25 21685607

[B30] TriNetX (2024). Linked EHR and claims. TriNetX. Available at: https://trinetx.com/real-world-data/linked/(Accessed May 3, 2024).

[B31] Wang L. Rastegar-Mojarad M. Ji Z. Liu S. Liu K. Moon S. (2018). Detecting pharmacovigilance signals combining electronic medical records with spontaneous reports: a case study of conventional disease-modifying antirheumatic drugs for rheumatoid arthritis. Front. Pharmacol. 9, 875. 10.3389/fphar.2018.00875 30131701 PMC6090179

[B32] Wang L. Rastegar-Mojarad M. Liu S. Zhang H. Liu H. (2017). Discovering adverse drug events combining spontaneous reports with electronic medical records: a case study of conventional DMARDs and biologics for rheumatoid arthritis. AMIA Jt. Summits Transl. Sci. Proc. 2017, 95–103. 28815115 PMC5543355

[B33] Wang S. V. Maro J. C. Gagne J. J. Patorno E. Kattinakere S. Stojanovic D. (2021). A general propensity score for signal identification using tree-based scan statistics. Am. J. Epidemiol. 190, 1424–1433. 10.1093/aje/kwab034 33615330

[B34] White R. W. Harpaz R. Shah N. H. DuMouchel W. Horvitz E. (2014). Toward enhanced pharmacovigilance using patient-generated data on the internet. Clin. Pharmacol. Ther. 96, 239–246. 10.1038/clpt.2014.77 24713590 PMC4111778

[B35] Yu Y. Ruddy K. J. Wen A. Zong N. Tsuji S. Chen J. (2020). Integrating electronic health record data into the ADEpedia-on-OHDSI platform for improved signal detection: a case study of immune-related adverse events. AMIA Jt. Summits Transl. Sci. Proc. 2020, 710–719. 32477694 PMC7233056

